# Disparate Modes of Evolution Shaped Modern Prion (*PRNP*) and Prion-Related Doppel (*PRND*) Variation in Domestic Cattle

**DOI:** 10.1371/journal.pone.0155924

**Published:** 2016-05-25

**Authors:** Brian W. Brunelle, Allison M. O’Grady, Eric M. Nicholson, Christopher M. Seabury

**Affiliations:** 1 Virus and Prion Research Unit, National Animal Disease Center, USDA, Agricultural Research Service, Ames, Iowa 50010, United States of America; 2 Department of Veterinary Pathobiology, College of Veterinary Medicine, Texas A&M University, College Station, Texas 77843-4467, United States of America; University of Maryland School of Medicine, UNITED STATES

## Abstract

Previous investigations aimed at determining whether the mammalian prion protein actually facilitates tangible molecular aspects of either a discrete or pleiotropic functional niche have been debated, especially given the apparent absence of overt behavioral or physiological phenotypes associated with several mammalian prion gene (*PRNP*) knockout experiments. Moreover, a previous evaluation of *PRNP* knockout cattle concluded that they were normal, suggesting that the bovine prion protein is physiologically dispensable. Herein, we examined the frequency and distribution of nucleotide sequence variation within the coding regions of bovine *PRNP* and the adjacent Doppel (*PRND*) gene, a proximal paralogue to *PRNP* on BTA13. Evaluation of *PRND* variation demonstrated that the gene does not depart from a strictly neutral model of molecular evolution, and would therefore not be expected to influence tests of selection within *PRNP*. Collectively, our analyses confirm that intense purifying selection is indeed occurring directly on bovine *PRNP*, which is indicative of a protein with an important role. These results suggest that the lack of observed fitness effects may not manifest in the controlled environmental conditions used to care for and raise *PRNP* knockout animals.

## Introduction

Transmissible spongiform encephalopathies (TSE) are a class of fatal neurodegenerative diseases that affect humans as well as livestock and wildlife in farmed and natural environments [1]. Human TSEs, such as Creutzfeldt-Jakob disease (CJD), variant CJD, Gerstmann Sträusler-Scheinker disease, and kuru are typically identified via observed clinical signs and post-mortem analyses [2]. Animal TSEs have largely been classified in the same manner and include transmissible mink encephalopathy, scrapie of sheep and goats, chronic wasting disease in free-ranging and captive species of Cervidae, feline spongiform encephalopathy, and bovine spongiform encephalopathy [2]. Importantly, the most profound unifying feature of these diseases is the accumulation of an infectious protease-resistant isoform (PrP^Sc^) of the host-encoded cellular prion protein (PrP^C^) within tissues of the central nervous system [1].

Notably, the prion protein gene (*PRNP*) is present in all vertebrate species [3], and the striking degree of amino acid conservation observed across a wide variety of highly divergent taxa suggests an important functional role for PrP^C^ [4]. Nevertheless, this evolutionary observation has been paradoxically shrouded by several knockout studies that failed to elucidate one or more overt physiological roles for PrP^C^ [5–7]. The implication that PrP^C^ deficient cattle may be considered safe mainstays for enhancing future agricultural products [8] is also in direct conflict with a previous investigation that provided unequivocal population and phylogenetic evidence for intense purifying selection constraining the long-term evolution of bovine *PRNP* [9]. Strong levels of purifying selection similar to that observed for bovine *PRNP* are normally only detected for genes encoding functionally important endogenous host proteins such as histones or ubiquitin [10, 11]. In the absence of function, what might explain such intense purifying selection?

The primary goal of this study was to determine whether strong purifying selection is acting directly on bovine *PRNP* [9], or if the selective signal actually radiates from a proximal bovine protein coding gene. Notably, the two proximal genes flanking bovine *PRNP* on BTA13 are *ZMYND11* (~202 kb upstream) and *PRND* (~31 kb downstream). *ZMYND11* encodes a transcriptional regulator protein that binds to adenovirus E1A proteins [12] while *PRND* encodes the doppel protein (Dpl), an evolutionarily related paralogue of *PRNP* believed to be involved in sperm maturation and capacitation [13]. Given the physical proximity between *PRNP* and *PRND* [14], as well as phylogenetic evidence that the two genes have been evolutionarily co-selected [15], we tested the hypothesis that strong selection within bovine *PRND* may be explanatory for selective signals previously detected in bovine *PRNP* [9]. Collectively, our analyses provide statistical support for intense purifying selection operating on bovine *PRNP*, with bovine *PRND* variation exhibiting no evidence for deviation from a strictly neutral model.

## Materials and Methods

### DNA panels

To comprehensively evaluate nucleotide sequence variation within bovine *PRNP* and *PRND*, we compiled and utilized data derived from 228 DNA samples previously employed to investigate bovine *PRNP* [16–18], including representatives of *Bos taurus taurus*, *Bos taurus indicus*, and their hybrids (composites). For *PRND* analysis, we used these same bovine samples, which included DNA extracted from 39 Holstein steers [17] and 189 commercially available spermatozoa samples of unrelated sires from the following 41 cattle breeds: Angus (4), Beefmaster (4), Belgian Blue (4), Blonde D’Aquitaine (5), Braford (4), Brahman (28), Brahmousin (2), Brangus (12), Braunvieh (5), Brown Swiss (4), Charolais (5), Chianina-Chiangus (5), Corriente (1), Gelbvieh (4), Gir (12), Guzerat (1), Hereford (3), Holstein (4), Limousin (3), Maine Anjou (4), Murray Gray (2), Nelore (8), Normande (1), Piedmontese (2), Red Angus (4), Red Brangus (1), Red Poll (1), Romagnola (2), Salers (3), Santa Gertrudis (9), Scottish Highland (1), Senepol (2), Shorthorn (19), Simbrah (3), Simmental (8), Tabapua (1), Tarentaise (1), Texas Longhorn (4), Three-way-cross (2), and White Park (1) [16, 18].

### *PRND* sequencing and multiple sequence alignments

Polymerase chain reaction primers amplifying a 991 bp product encompassing the entire *PRND* coding region were designed from the Genbank reference sequence DQ205538 (5'-AGATCACTATCCTGAATGGTG-3', 5'-TTTAGGTAGAGCCTGGAGAG-3'). Each 25-μL PCR reaction contained 50 ng of genomic DNA, 1x PCR buffer with 1.5 mM MgCl_2_, 1 mM each dNTP, 0.8 μM of each primer, and 1.5 units of Taq polymerase. Amplification conditions were as follows: 94°C for 1 min; 35 cycles of 94°C for 30s, 56°C for 30s, 72°C for 30s; 72°C for 2 min; 4°C hold. PCR products were visualized and verified on 2% NuSieve gels (Cambrex, Rockland, ME) and subsequently treated with ExoSAP-IT (GE Healthcare, Piscataway, NJ) for purification. Purified *PRND* PCR products were directly sequenced using the amplification primers and a pair of internal primers (5'-TTGCCAAGTACCTCCCAG-3', 5'-TTTCCTTGGTGACATTGG-3') in conjunction with standard dye terminator cycle sequencing technology. Individual *PRND* contig sequences were assembled for each sample using Lasergene 6 (DNASTAR, Inc., Madison, WI). Thereafter, *PRND* sequences were aligned using ClustalX [19] and submitted to GenBank (accession numbers JF808218-JF808446). Likewise, bovine *PRNP* exon 3 sequences [9, 16, 17] were also aligned using ClustalX [19] as previously described [9].

### Haplotype inference and network analysis

For haplotypes that were not phase-resolved through a second round of PCR, cloning, and bidirectional sequencing [9], we assembled unphased diploid genotypes for nucleotide sequence variation observed within the coding regions of bovine *PRNP* and *PRND*, including both single nucleotide polymorphisms (SNPs) and insertion-deletion mutations (indels). Bovine *PRNP* and *PRND* haplotype reconstructions were performed with PHASE 2.1 [20, 21] using all intragenic polymorphisms, all cattle (n = 228), and the—X10 option as previously described [22]. Haplotype phases previously established for 112 of the *PRNP* samples [9] were designated as phase-known for haplotype reconstruction.

Median joining haplotype networks for bovine *PRNP* and *PRND* were constructed using the program Network 4.5.1.0 (Fluxus Technology Ltd) in conjunction with the suggested character weights of 10 for SNPs and 20 for indels. Network branch angles were adjusted to ensure clarity without modifying branch lengths.

### Sequence analysis

Phased *PRNP* and *PRND* sequences were used in conjunction with the software program DnaSP 5.1 [23] to estimate the number of potentially synonymous and non-synonymous nucleotide sites, the number of synonymous and non-synonymous polymorphisms, and the number of synonymous (*d*_S_) and non-synonymous (*d*_N_) substitutions per site with Jukes-Cantor correction [24, 25]. Of the 456 phased resolved *PRNP* sequences evaluated, 19 had alignment gaps in the octapeptide repeat region and were excluded from the synonymous and non-synonymous analysis.

### Tests of selection

To evaluate potential deviations from a strictly neutral model of molecular evolution, we used the Z-test implemented in MEGA 4 [26] to evaluate the null hypothesis that *d*_N_ = *d*_S_ (strict neutrality; two-tailed test) and the research hypothesis *d*_N_ < *d*_S_ (one-tailed test) using pair-wise deletion of alignment gaps with Jukes-Cantor correction. For pairwise tests of selection, we estimated the variance of (*d*_N_ − *d*_S_) via bootstrap analysis with 1000 replicates. Frequency distribution tests, including Tajima’s D [27] and Fu and Li’s Tests (D*and F*) [28], were performed in DnaSP v5.1 [23] using all *PRNP* and *PRND* coding region polymorphisms (excluding gaps). Significance was assessed for each test by estimating confidence intervals through coalescent simulations using the observed number of segregating sites with 5,000 replicates [23]. All tests were conducted both with and without the 19 *PRNP* haplotypes possessing alignment gaps. The program GARD (Genetic Algorithm for Recombination Detection) was used to detect the presence of recombination in the *PRND* and *PRNP* sequences [29]. No overt evidence of recombination was detected. The potential for episodic and pervasive selective pressures was estimated for the unique haplotypes using the tree-based analysis programs MEME (Mixed-Effects Model of Evolution [30]), FUBAR (Fast, Unconstrained Bayesian AppRoximation [31]), and BS-REL (Branch-site Random-effects Likelihood [30]), which are part of the HyPhy software suite [32], as executed in the Datamonkey webserver [33]. Chi-square with Yates correction was used to assess the overall magnitude of differences in the distributions of polymorphisms at synonymous and non-synonymous sites with respect to *PRNP* and *PRND* (http://www.quantpsy.org/chisq/chisq.htm).

## Results

### General nucleotide data

To facilitate a detailed comparative analysis between bovine *PRNP* and *PRND* variation, we computed the number of potentially synonymous and non-synonymous nucleotide sites [24, 25], the total number of synonymous and non-synonymous SNPs, and the number of synonymous and non-synonymous substitutions per site (Table 1). The coding sequence of bovine Doppel is 537 bp in length, and 6 SNPs were identified among the 228 samples at positions 141(A/G), 149(A/G), 172(A/G), 285(C/T), 395(A/G), and 528(A/T). The three SNPs detected at sites 141, 285, and 528 were predicted to encode synonymous substitutions, while SNPs at positions 149, 172, and 395 were predicted to encode amino acid replacements (R50H, A58T, Q132R, respectively; IUB/IUPAC Amino Acid Codes). All 6 *PRND* polymorphisms were detected among samples representing *B*. *t*. *taurus* and the composite cattle, but only 3 were predicted in samples representing *B*. *t*. *indicus* cattle (141, 172, 395). Collectively, 30 synonymous SNPs and one non-synonymous SNP (S154N; *B*. *t*. *indicus* and composite cattle) were predicted in the coding sequences of bovine *PRNP*, as previously described [9].

**Table 1:** Nucleotide data for *PRND* and *PRNP* genes.

	#alleles	#syn sites ^a^	# syn mut ^b^	# non-syn sites ^c^	# non-syn mut ^d^	*d*_S_ ^e^	*d*_N_ ^f^	*d*_N_*/d*_S_
***PRND***								
*B*. *t*. *taurus*	278	119.06	3	414.94	3	0.0036	0.0013	0.3705
*B*. *t*. *indicus*	100	119.36	1	414.64	2	0.0028	0.0024	0.8514
Composite	78	119.12	3	414.88	3	0.0043	0.0017	0.3869
Total	456	119.14	3	414.86	3	0.0037	0.0018	0.4796
***PRNP***								
*B*. *t*. *taurus*	264	186.33	23	605.67	0	0.0064	0.0000	0.0000
*B*. *t*. *indicus*	98	186.33	10	605.67	1	0.0102	0.0003	0.0255
Composite	75	186.33	10	605.67	1	0.0070	0.0001	0.0128
Total	437	186.33	30	605.67	1	0.0076	0.0001	0.0105

^a^ number of potentially synonymous sites

^b^ number of synonymous mutations observed

^c^ number of potentially non-synonymous sites

^d^ number of non-synonymous mutations observed

^e^ synonymous substitutions per site

^f^ non-synonymous substitutions per site

### Haplotype data

**Fig 1:**
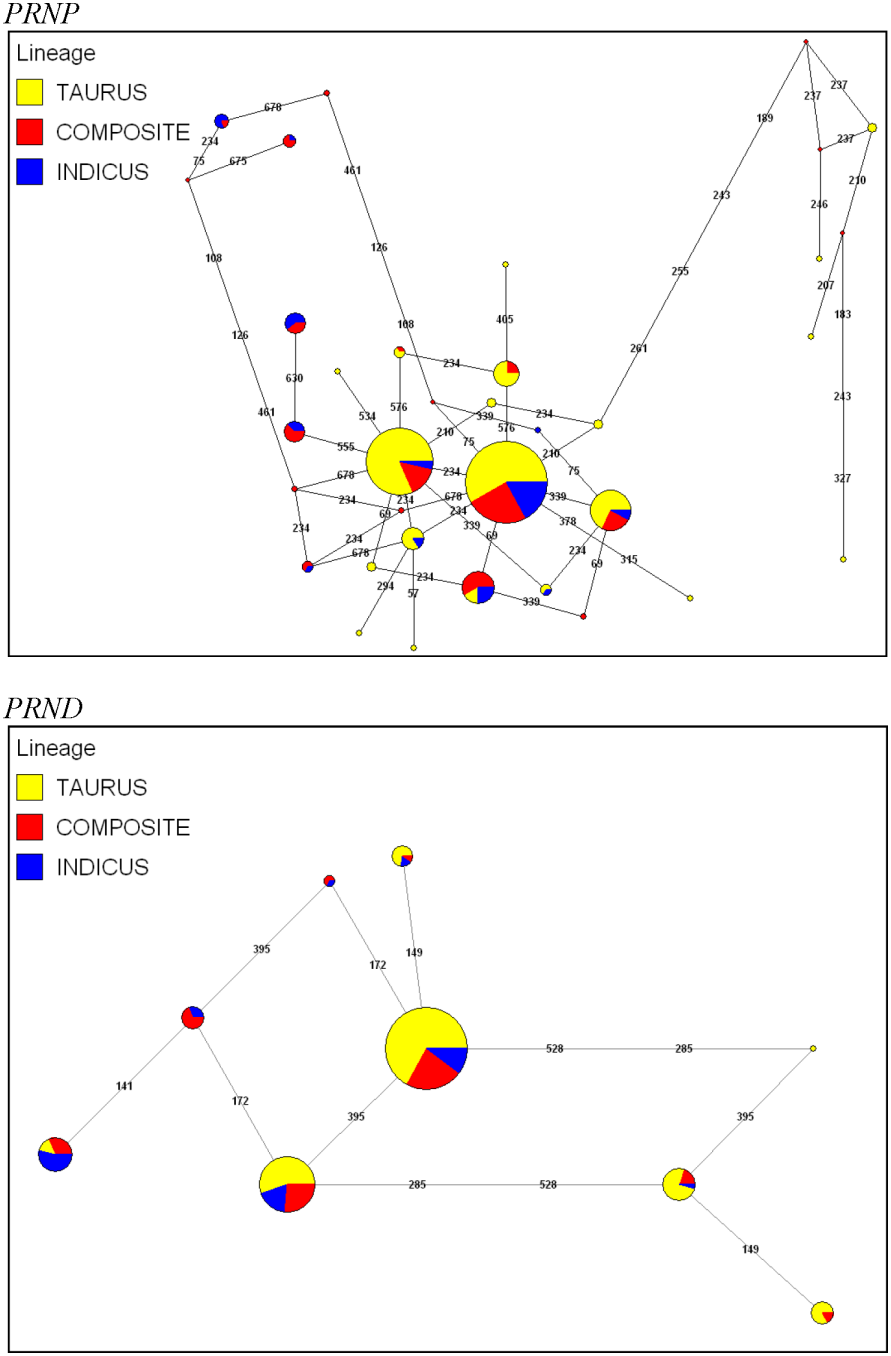
Median joining haplotype networks for *PRNP* and *PRND*. Median joining haplotype networks were constructed for bovine *PRNP* and *PRND* using character weights of 10 for SNPs and 20 for indels. Network branch angles were adjusted to ensure clarity without modifying branch lengths.

The 31 polymorphic nucleotide sites in *PRNP* yielded 31 distinct haplotypes (S1 Table), with corresponding frequency distributions among *B*. *t*. *taurus*, *B*. *t*. *indicus*, and composite cattle that were similar to a previous *PRNP* analysis [16, 18]. The 6 polymorphic nucleotide sites in *PRND* produced 9 individual haplotypes (Table 2). *PRND* haplotypes #1 and #2 accounted for the majority of all haplotypes predicted in *B*. *t*. *taurus* (85%), *B*. *t*. *indicus* (63%), and composite (79%) cattle. Notably, four *PRND* haplotypes were exclusive to *B*. *t*. *taurus* in our samples, which resulted from 3 putative SNPs that were not detected among *B*. *t*. *indicus* cattle. Interestingly, haplotypes #5, #6, and #9 were identified primarily in *B*. *t*. *indicus* (37%) compared to *B*. *t*. *taurus* (1%; See Table 1). Median joining haplotype networks constructed as putative representations of bovine *PRNP* and *PRND* evolution (Fig 1) provide evidence for only a few major haplotypes as well as haplotype sharing across the three investigated bovine lineages (*B*. *t*. *taurus*, *B*. *t*. *indicus*, and composites). Moreover, specialized beef and dairy breeds could not be differentiated based on *PRNP* or *PRND* haplotypes, which is concordant with a recent study on bovine Toll-like receptor evolution [34].

**Table 2:** *PRND* haplotype data.

hap# ^a^	141 ^b^	149 ^b^	172 ^b^	285 ^b^	395 ^b^	528 ^b^	% *B*. *t*. *taurus*	*% B*. *t*. *indicus*	% composite
1	G	G	G	C	A	T	69.49	43.88	62.50
2	G	G	G	C	G	T	15.07	19.39	16.67
3	G	G	G	T	A	A	0.37	0.00	0.00
4	G	G	G	T	G	A	6.99	0.00	8.33
5	G	G	A	C	A	T	0.00	3.06	0.00
6	G	G	A	C	G	T	0.00	13.27	0.00
7	G	A	G	C	A	T	2.94	0.00	4.17
8	G	A	G	T	G	A	3.68	0.00	2.78
9	A	G	A	C	G	T	1.47	20.41	5.56

^a^ haplotype identifier

^b^ nucleotide position in *PRND*

### Tests of selection

The observed ratio of synonymous to non-synonymous polymorphisms predicted for bovine *PRNP* was highly skewed (30:1, respectively), whereas *PRND* exhibited no skewness (3:3, respectively). To further assess and compare the potential for functional and/or selective constraint(s) acting on bovine *PRNP* and *PRND*, we computed Tajima's D, Fu and Li’s D*, and Fu and Li’s F* (Table 3). All frequency distribution tests indicated that variation within bovine *PRNP* does not adhere to a strictly neutral model, which is concordant with a previous study demonstrating that bovine *PRNP* is subject to strong purifying selection [16]. In contrast, frequency distribution tests carried out for *PRND* revealed no evidence for departure from a strictly neutral model (Table 3).

**Table 3:** Tests of selection for *PRND* and *PRNP* genes.

	Fu and Li D*	Fu and Li F*	Tajima's D	Z-test *d*_N_ = *d*_S_	Z-test *d*_N_ < *d*_S_
***PRND***					
*B*. *t*. *taurus*	1.04	0.84	0.07	-0.871	0.917
*B*. *t*. *indicus*	0.83	1.48	2.21	-0.117	0.116
Composite	1.14	0.84	-0.16	-0.899	0.938
All Cattle	1.00	1.01	0.55	-0.800	0.824
***PRNP***					
*B*. *t*. *taurus*	-4.89 ^a^	-4.53 ^a^	-2.00 ^a^	-2.374 ^a^	2.435 ^a^
*B*. *t*. *indicus*	0.67	0.32	-0.47	-2.656 ^a^	2.756 ^a^
Composite	0.04	-0.54	-1.39	-2.341 ^a^	2.416 ^a^
All Cattle	-3.64 ^a^	-3.55 ^a^	-1.95 ^a^	-2.753 ^a^	2.797 ^a^

^a^ Statistically significant (*P* < 0.05)

The Z-test was conducted to determine if the rate of change between synonymous sites and non-synonymous sites was strictly neutral (*d*_N_ = *d*_S_) or directional (*d*_N_ < *d*_S_) for *PRND* and *PRNP* (Table 3). Variation within bovine *PRND* did not deviate from strict neutrality (Table 3). However, a significant deviation was noted for variation within bovine *PRNP*, and was consistent with purifying selection [9]. Though the direction of selection cannot accurately be determined within a population by the absolute value of the *d*_N_/*d*_S_ ratio relative to one [35], differences in the magnitude of selective constraint(s) between the two genes may still be highlighted by the *d*_N_/*d*_S_ ratio (i.e., *PRND* = 0.4796; *PRNP* = 0.0105). Moreover, using the chi-square test for non-integers with Yate's correction for continuity (two-tailed) to compare the number of synonymous polymorphisms and potentially synonymous nucleotide sites between *PRNP* and *PRND* revealed a significant difference between the two genes (*P* = 0.005), whereas the rate of nonsynonymous change was not found to differ (*P* = 0.413). The difference in synonymous changes, but not nonsynonymous changes, further supports the supposition that bovine *PRNP* is under purifying selection, with selective signals that cannot be attributed to *PRND*.

To further clarify inferences drawn from our initial tests of selection, we also sought to investigate the potential for episodic selection within bovine *PRND* and *PRNP*. Analyses performed using the programs MEME and BS-REL [30] failed to detect evidence for episodic selection within *PRND* and *PRNP* (S1 File). Moreover, using the program FUBAR [31], we observed site-specific evidence for pervasive purifying selection within both *PRND* (n = 3 sites/codons) and *PRNP* (n = 9 sites/codons); whereas evidence for pervasive diversifying selection was only detected in *PRND* (n = 2 sites/codons; S1 File). Collectively, these analyses further support the conclusion that nearly all nucleotide sites within the coding sequence of bovine *PRND* adhere to a strictly neutral model of molecular evolution, whereas the coding sequence of bovine *PRNP* is subject to intense purifying selection, thereby suggesting a potentially important role for bovine PrP.

## Discussion

Herein, we have demonstrated that strong purifying selection on bovine *PRNP* cannot be attributed to selective pressures that are acting on a neighboring coding region (i.e., *PRND*), as bovine *PRND* variation does not depart from a strictly neutral model of molecular evolution. We also show that *B*. *t*. *taurus* and *B*. *t*. *indicus* share the major haplotypes for both genes, thereby suggesting that the majority of the polymorphisms, and the differences in selective constraints between *PRNP* and *PRND*, likely occurred before taurine and indicine divergence.

Notably, we cannot dispute the fact that prion knockouts analyzed to date lack gross evidence of deleterious effects [36]. However, we do dispute the conclusion that a lack of deleterious effects suggests that *PRNP* is simply dispensable [5], as biological dispensability, the manifestation of disease, and evolutionary rate have a significant relationship [37–39]. Interestingly, prior analyses of essential genes, Mendelian disease genes, and complex disease genes, as compared to non-essential and non-disease genes, demonstrate evidence of strong purifying selection [38, 39]. Relevant to this study, a protein under intense purifying selection (i.e., bovine *PRNP*), with even a small but measureable fitness effect, may be essential for the functional viability of certain cells and their corresponding tissues [37, 40–42]. For this reason, it should be noted that fitness effects may simply not have manifested in the controlled environmental conditions utilized for prion knockout cattle, or perhaps they were not recognizable through the limited assessment of these animals [5]. Examples exist in the literature of gene knock out effects observed to act only in a gender specific manner or in an age-dependent manner [43–46]. *PRNP* knockout mice have in fact been shown to have numerous subtle phenotypic differences [36]. Other than resistance to TSE, the first documented phenotypic change in PrP knockout mice was disturbances in sleep and altered circadian rhythms, as compared to wild-type mice, when housed in constant darkness [47, 48]. The knock out cattle used as evidence to suggest that *PRNP* is a dispensable gene were all castrated males studied as mature adults with no physiological or immunological stress placed on the animal(s). In general, given many accounts of gender-specific and age-specific effects observed for gene knockouts, as well as specific examples for *PRNP* reviewed by Steele and colleagues [36], it is possible that no gross changes were observed for castrated males under the carefully controlled environments (i.e., feeding and housing) used for the knock out cattle studies. These studies lack the exhaustive assessment of all developmental and physiological conditions necessary to assert any claims as to the biological dispensability of *PRNP*.

Fitness effect(s) or dispensability is not the only contribution to evolutionary rate. The number of protein-protein interactions has a strong correlation to both evolutionary rate and fitness [37]. Evidence that 30 different proteins likely interact with PrP [49], could at least, in part, explain the high degree of conservation commonly observed for the mammalian *PRNP* gene. Putative functional roles for PrP are likewise multifaceted with numerous cellular pathways influenced by PrP [50–52]. PrP has been shown to have a role in epithelial to mesenchymal transition [53], and several studies report protective roles for PrP, including protection against oxidative stress [54, 55]. PrP may also play a direct role in Alzheimer’s disease, another neurodegenerative disorder [54, 56]. High levels of PrP expression are found in placenta, indicating a potential role in reproduction [57]; this role ties closely with evidence that Dpl and a third member of the prion gene family, Shadoo (Sho), can both interact with PrP, as well as function in place of PrP in aspects of reproduction [58]. Consistent with this is the observation that in the absence of PrP, aberrant expression of Dpl in the CNS results in mice that develop ataxia due to apoptosis of cerebellar cells [59], suggesting that Dpl is interacting with ligands that would normally interact with PrP. Expression of PrP in these tissues is sufficient to negate this effect [60]. Sho exhibits PrP-like neuroprotective properties with regard to Dpl-induced neurotoxicity in the CNS [49], indicating that Sho is also likely to be capable of interacting with the same ligands. A clear overlap of interactions exists between the various members of the prion gene family, which may be one plausible explanation for the lack of a discernable phenotype in *PRNP* knockout animals.

## Supporting Information

S1 File BSREL and FUBAR analysis output. (DOCX)

S1 Table *PRNP* haplotype information. (XLSX)
