# Supplementary material for: Disparate Modes of Evolution Shaped Modern Prion (PRNP) and Prion-Related Doppel (PRND) Variation in Domestic Cattle
Source: PLoS One. 2016 May 25;11(5):e0155924. doi: 10.1371/journal.pone.0155924 (PMC4880211; doi:10.1371/journal.pone.0155924)
Supplement: S1 File — (DOCX) [file pone.0155924.s001.docx]

**S2 File. BSREL and FUBAR analysis output**

**BS-REL *PRND* output.** BS-REL was performed for *PRND*. The colors denote selection strength (red corresponds to ω > 5, grey corresponds to ω=1, and blue corresponds to ω = 0).

**BS-REL *PRNP* output.** BS-REL was performed for *PRNP*. The colors denote selection strength (red corresponds to ω > 5, grey corresponds to ω=1, and blue corresponds to ω = 0).

**FUBAR *PRND* output**. FUBAR was used to evaluate *PRND*.

Found **2** sites with evidence of pervasive diversifying selection at posterior probability ≥0.9.

| Codon | α | β | β-α | Posterior Prob β>α | Emp. Bayes Factor | PSRF | N_eff_ |
| --- | --- | --- | --- | --- | --- | --- | --- |
| 50 | 1.00973 | 6.56993 | 5.5602 | 0.909865 | 12.2157 | 0.998061 | 1626.04 |
| 132 | 0.734408 | 8.2755 | 7.54109 | 0.959772 | 28.8719 | 0.997271 | 2186.47 |

Found **3** sites with evidence of pervasive purifying selection at posterior probability ≥0.9.

| Codon | α | β | β-α | Posterior Prob β<α |
| --- | --- | --- | --- | --- |
| 47 | 5.15237 | 0.366814 | -4.78556 | 0.914462 |
| 95 | 6.00412 | 0.579445 | -5.42467 | 0.91369 |
| 176 | 5.80424 | 0.294673 | -5.50957 | 0.938313 |

**FUBAR *PRNP* output**. FUBAR was used to evaluate *PRNP*.

Found no sites with evidence of pervasive diversifying selection at posterior probability ≥0.9.

Found **9** sites with evidence of pervasive purifying selection at posterior probability ≥0.9.

| Codon | α | β | β-α | Posterior Prob β<α |
| --- | --- | --- | --- | --- |
| 25 | 8.67109 | 0.180477 | -8.49061 | 0.994183 |
| 69 | 3.65185 | 0.204008 | -3.44784 | 0.958255 |
| 70 | 8.51079 | 0.218766 | -8.29203 | 0.99142 |
| 77 | 3.49564 | 0.213917 | -3.28172 | 0.955885 |
| 78 | 13.9906 | 0.241384 | -13.7492 | 0.999892 |
| 79 | 2.44931 | 0.157319 | -2.29199 | 0.950605 |
| 81 | 4.00213 | 0.212558 | -3.78957 | 0.964146 |
| 113 | 2.8773 | 0.156257 | -2.72104 | 0.958183 |
| 226 | 2.11604 | 0.155398 | -1.96064 | 0.942178 |
